# Effect of MK-801 and Clozapine on the Proteome of Cultured Human Oligodendrocytes

**DOI:** 10.3389/fncel.2016.00052

**Published:** 2016-03-03

**Authors:** Juliana S. Cassoli, Keiko Iwata, Johann Steiner, Paul C. Guest, Christoph W. Turck, Juliana M. Nascimento, Daniel Martins-de-Souza

**Affiliations:** ^1^Laboratory of Neuroproteomics, Department of Biochemistry and Tissue Biology, Institute of Biology, University of CampinasCampinas, Brazil; ^2^United Graduate School of Child Development, Department of Development of Functional Brain Activities, Research Center for Child Mental Development, Hamamatsu University School of Medicine, Osaka University and Kanazawa University and Chiba University and University of FukuiFukui, Japan; ^3^Department of Psychiatry, University of MagdeburgMagdeburg, Germany; ^4^Department of Translational Research in Psychiatry, Max Planck Institute of PsychiatryMunich, Germany; ^5^D’Or Institute for Research and Education Rio de Janeiro, Brazil; ^6^UNICAMP Neurobiology CenterCampinas, Brazil

**Keywords:** glial cells, oligodendrocyte, proteomics, schizophrenia, pharmacology, clozapine, MK801

## Abstract

Separate lines of evidence have demonstrated the involvement of *N*-methyl-D-aspartate (NMDA) receptor and oligodendrocyte dysfunctions in schizophrenia. Here, we have carried out shotgun mass spectrometry proteome analysis of oligodendrocytes treated with the NMDA receptor antagonist MK-801 to gain potential insights into these effects at the molecular level. The MK-801 treatment led to alterations in the levels of 68 proteins, which are associated with seven distinct biological processes. Most of these proteins are involved in energy metabolism and many have been found to be dysregulated in previous proteomic studies of post-mortem brain tissues from schizophrenia patients. Finally, addition of the antipsychotic clozapine to MK-801-treated oligodendrocyte cultures resulted in changes in the levels of 45 proteins and treatment with clozapine alone altered 122 proteins and many of these showed opposite changes to the MK-801 effects. Therefore, these proteins and the associated energy metabolism pathways should be explored as potential biomarkers of antipsychotic efficacy. In conclusion, MK-801 treatment of oligodendrocytes may provide a useful model for testing the efficacy of novel treatment approaches.

## Introduction

The *N*-methyl-D-aspartate recept or (NMDAr) is an ionotropic receptor activated by glutamate, allowing the non-selective influx of calcium and sodium and outflow of potassium. The function of NMDAr in neurons is well-described as it participates actively on glutamatergic transmission, which is known to be defective in schizophrenia and related psychiatric disorders.

Schizophrenia presents with a variety of both positive and negative symptoms (Lewis, 2000). There are also disturbances in cognitive processes, such as attention and working memory, which can appear prior to the onset of the clinical condition. These cognitive problems represent core features of the illness and are associated to NMDAr dysfunction (Hahn et al., 2006). NMDAr antagonists exacerbate pre-existing symptoms in patients with schizophrenia and may drive schizophrenia-like symptoms in healthy mice and human volunteers (Gunduz-Bruce, 2009). Moreover, these antagonists have been found to trigger sensory and motor disturbances in rats, similar to those displayed by patients with schizophrenia (Kovacic and Somanathan, 2010). At the cellular level, treatment with NMDAr antagonists have been shown to cause neuronal degeneration in retrosplenial, pyriform, and entorhinal cortices, as well as the *tenia tecti amygdalae* (Horváth et al., 1997).

While NMDAr function has been well-described in neurons, its function in glial cells such as astrocytes and oligodendrocytes still needs clarification despite intensive investigation over the past 10 years (Salter and Fern, 2005; Cao and Yao, 2013). This is likely to provide further insights into the pathways affected in schizophrenia, given the role of oligodendrocytes in the establishment and course of the disease (Cassoli et al., 2015). Cultured oligodendrocytes present glutamate-responsiveness to NMDAr, and may even release glutamate in certain conditions (Deng et al., 2003). This enables the study of the molecular mechanisms of oligodendrocytes *in vitro*.

One way of modulating NMDAr function in experimental settings is by employing pharmacological interventions. MK-801 (or [5R,10S]-[+]-5-methyl-10,11-dihydro-5*H*-dibenzo[*a,d*]cyclohepten-5,10-imine or Dizocilpine) is a compound that belongs to the secondary bicyclic amine class and acts as non-competitive NMDAr antagonist, not only in neurons, but also in oligodendrocytes (Li et al., 2013). This compound binds to two sites on the NMDAr-ion channel complex in a manner similar to phencyclidine (PCP; Kornhuber et al., 1989) *in vivo* and *in vitro* (Murray et al., 2000). The NMDAr mediates glutamatergic transmission and plays a key role in neural plasticity of central nervous system (CNS; Harrison and Weinberger, 2004).

Besides the neuroprotective effects observed in stroke, trauma, Parkinson and organophosphate-induced seizure models, MK-801 also induces schizophrenia-like symptoms (Kovacic and Somanathan, 2010) such as alterations in prepulse inhibition (PPI; Zangrando et al., 2013). This has led researchers to employ MK-801 as a pharmacological model of schizophrenia (Paulson et al., 2007) for testing effects of antipsychotics such as clozapine (Paulson et al., 2007; Zuo et al., 2009; Vardigan et al., 2010; Brown et al., 2014).

Previous studies have shown that the NMDAr is also present in oligodendrocytes (Simon et al., 1984; Karadottir et al., 2005; Salter and Fern, 2005; Micu et al., 2006) and may be involved in regulation of myelination processes (Li et al., 2013). NMDA receptor signaling in oligodendrocytes also plays a crucial role in their energy metabolism and regulates differentiation and migration of these cells (for review see (Cao and Yao, 2013). Here, we have carried out a quantitative proteomic analysis to analyze protein expression changes in the human oligodendrocyte hybrid cell line (MO3.13) following treatment with MK-801 or the antipsychotic clozapine compared to control cells. The main objective was to shed light on the biochemical mechanisms involving NMDAr function in oligodendrocytes in order to determine whether these cells could be useful in future studies to model some aspects of schizophrenia.

## Experimental Procedures

### Cell Cultures, Treatments, and Proteome Extraction

**FIGURE 1:**
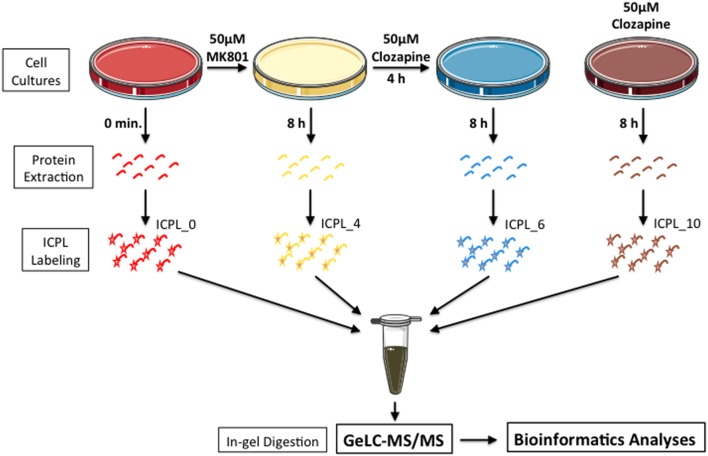
**Experimental setup.** Briefly, MO3.13 cells were grown in the presence of MK-801 or clozapine or MK-801/clozapine. Proteins were extracted, labeled with ICPL and mixed with the same peptide amount. To reduce the proteome complexity, the samples were prior fractionated by SDS-PAGE, and protein bands were digested by trypsin at room temperature. In last step, the proteins from each gel slice were then subjected to LC-MS/MS analyses.

MO3.13 cells were maintained in DMEM medium supplemented with 2 mM L-glutamine, 1% penicillin/streptomicyn (Sigma-Aldrich, St. Louis, MO, USA) and 10% heat-inactivated fetal bovine serum (Life Technologies, Darmstadt, Germany), at 37°C in humidified atmosphere containing 5% CO_2_, as described previously (Iwata et al., 2013). Cells were treated once and collected after 8 h as follows: Group 1 – 50 mM MK-801; Group 2 – 50 mM MK-801 plus 50 mM clozapine after 4 h; Group 3 – 50 mM clozapine; Group 4 – vehicle solution (0.01 M HCl; **Figure 1**). The glycine (0.4 mM) and glutamate (20 uM) contained in DMEM and FBS respectively are sufficiently high to activate NMDA receptors (Blanke and VanDongen, 2009; Cummings and Popescu, 2015).

MO3.13 cells were centrifuged at 1,000 *g* for 3 min and the pellets homogenized in 50 μL of 7 M urea, 2 M thiourea, 4% CHAPS, 2% ASB-14, and 70 mM DTT using a sample grinding kit (GE Healthcare, Uppsala, Sweden; Martins-de-Souza et al., 2007). Protein lysates were centrifuged for 10 min at 13,800 *g*, the supernatants collected and protein concentrations determined by Bradford assay (Bio-Rad, Munich, Germany).

### ICPL (Isotope-Coded Protein Labeling) Labeling

Cell lysate proteins were labeled with ICPL reagent from SERVA ICPL^TM^ Quadruplex Kit (SERVA Electrophoresis, Heidelberg, Germany), as previously described (Maccarrone et al., 2014). Equal amounts of light and heavy labeled samples were combined and separated by 12% SDS gel electrophoresis before brilliant Coomassie staining. After staining, each gel lane was sliced in 10 pieces and the protein bands were digested in-gel using a 1:80 ratio of trypsin:ammonium bicarbonate. The resulting peptides were dried and stored at -80°C prior to shotgun mass spectrometry analyses.

### NanoLC-ESI MS/MS, Data Processing and Database Searching

Extracted peptides were dissolved in 0.1% formic acid aqueous solution and analyzed using a 2D-nano-LC system (Eksigent, Dublin, CA, USA) coupled online to an LTQ-Orbitrap XL mass spectrometer (Thermo Scientific, Bremen, Germany), as previously described (Maccarrone et al., 2013). The MS/MS fragmentation spectra were acquired in data dependent mode, and the five most intense signal ions (m/z) in each scan were selected for fragmentation. Nanoflow LC–MS/MS was performed in automatic mode via Xcalibur software (version 2.0.7, Thermo Scientific, San José, CA, USA). Each gel slice generated one MS raw data file, which was processed for generation of the.mgf file. The target and decoy (reverse sequence) databases were searched using the UniProt human protein database (release 2013 08, 20,266 sequences) through the Mascot server. The search parameters were (1) peptide and fragment ion mass accuracy 10 ppm and 0.5 Da, respectively, (2) protein and peptide FDRs 1%, (3) two missed cleavages, (4) trypsin as enzyme, (5) fixed modification = cysteine carbamidomethylation and variable modification = methionine oxidation and ICPL labeling.

### Proteome Quantification

Identified proteins had to fit the following criteria in all analyzed datasets to be considered for quantification as described previously (Maccarrone et al., 2014): (1) identification by at least 2 non-redundant peptides; (2) fold changes not greater than ±15; and (3) the standard deviation of quantified peptides not greater than 10. Determination of isotope-labeled peptide ratios was performed with MASCOT Distiller (Matrix Sciences). The software calculates the fold-changes of each identified peptide, considering the signal intensities of the same peptide across different samples. The fold changes of all peptides of a given protein were averaged, thereby determining the protein fold change. Based on our previous results (Maccarrone et al., 2014), we only considered proteins differentially expressed if they presented a fold-change greater than ±1.5 and proteins with fold changes between 1.5 and 2 were only considered if quantified by at least 5 peptides. In addition, all quantitated proteins were required to have a normal distribution at the peptide level, as determined by the Shapiro–Wilk *W*-test so an analysis of variance (ANOVA) could be employed to determine differentially expressed proteins. Only those with *p*-values lower than 0.05 were considered further.

### Pathway and Functional Correlation Analysis

The Uniprot accession codes of differentially expressed proteins were mapped to Gene Ontology (GO) categories (biological function and molecular process), using a script linked to the Human Protein Reference Database^1^. The same codes were also uploaded into the QIAGEN Ingenuity^®^ Pathway Analysis software (IPA^®^ , QIAGEN, Redwood City, CA, USA^2^), to the associated over-represented biological pathways and functions. IPA core analysis was performed using experimentally observed data from human and CNS cell lines, with canonical pathways, diseases and biological functions, and networks explored in detail. The refinement of the network generated by IPA was performed applying the following parameters: (1) direct/indirect interactions, (2) experimentally observed as confidence level, (3) human as species, (4) tissues/cell lines, and (5) disease related to the CNS.

### Western Blot

**FIGURE 2:**
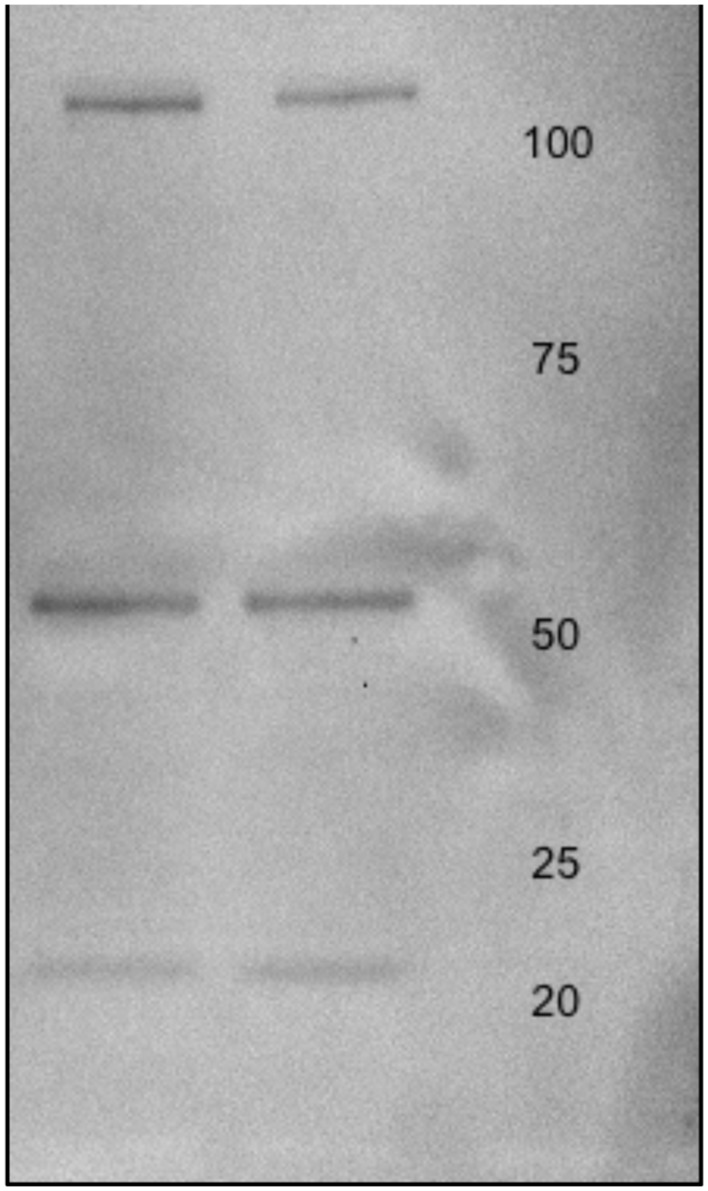
***N*-methyl-D-aspartate (NMDA) receptor Western Blot in MO3.13 protein lysates**.

MO3.13 protein lysates were (20 μg) were electrophoresed on 12% sodium dodecyl sulfate (SDS) minigels prepared in house. Proteins were transferred to Immobilon-FL polyvinyldiphenyl fluoride (PVDF) membranes (Millipore; Bedford, MA, USA) at 100 V for 1 h using a cooling system. PVDF membranes were then treated with 5% Carnation instant non-fat dry milk powder in Tris buffered saline (pH 7.4) containing 0.1% Tween -20 (TBS-T) for 4 h and rinsed in TBS-T three times for a total of 20 min. Membranes were incubated with Anti-Glutamate Receptor NMDAR1 (NR1) antibody produced in rabbit at a dilution of 1:000 in TBS-T overnight at 4°C (Sigma-Aldrich; Taufkirchen, Germany). Membranes were then washed twice with TBS-T for 15 min per wash. Next, the membranes were incubated with anti-c-MYC-peroxidase antibody (GE Healthcare; Uppsala, Sweden) for 40 min at room temperature, washed with water and TBS-T, and incubated with enhanced chemiluminescence (ECL) solution (GE Healthcare) for 1 min. The membranes were scanned using a Gel Doc^TM^ XR+ System (Silk Scientific Incorporated; Orem, UT, USA) and the optical densities of the immunoreactive bands were measured using Quantity One software (Bio-Rad; **Figure 2**).

## Results

**FIGURE 3:**
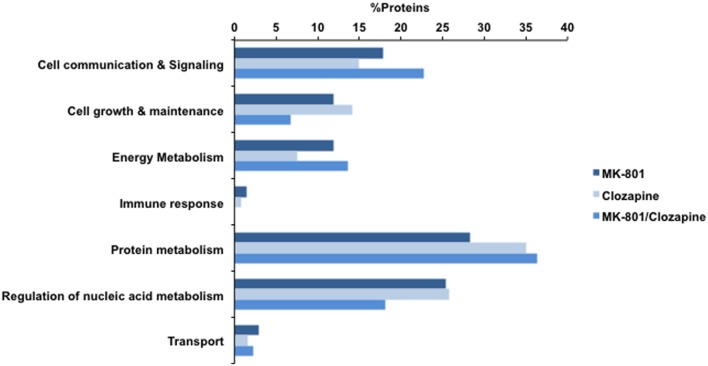
**Biological processes involving the proteins identified with MK-801, Clozapine, and concomitant (MK-801 and Clozapine) treatment.** Bars in dark blue, blue, and light blue depict MK-801, Clozapine and MK-801/Clozapine treatments, respectively.

All treatments here studied (MK-801, MK-801+Clozapine and only clozapine) modified the proteome of cultured oligodendrocytes. The differentially expressed proteins were analyzed in terms of their biological processes, and the result is shown in **Figure 3**. All three analyzed proteomes presented differences in similar biological processes. For instance, proteins associated to protein metabolism were the most prevalent on all three different treatments in percentage terms. On the other hand, there are proteins and functional correlations, which are specifically modulated by each different treatment analyzed.

### MK-801-Treated Oligodendrocytes

MK-801 treatment for 8 h induced changes in the levels of 68 proteins in cultured oligodendrocytes (**Supplementary Table S1**). These proteins are mostly associated with energy metabolism functions and included aldolase A (ALDOA), aldolase C (ALDOC), malate dehydrogenase (MDH2), and transketolase (TKT; **Table 1**). In addition, proteins such as prohibitin (PHB) and annexin 5 (ANXA5), which are associated with communication and cell signaling, and the protein metabolism-related proteins nucleophosmin (NPM1), 40S ribosomal protein S16 (RPS16) and 60S ribosomal protein L7A (RPL7A), were specifically altered by the MK-801 treatment.

**Table 1:** Comparison of proteomic changes in oligodendrocytes (MO3.13 cells) after the indicated drug treatments.

Symbol	Description	Fold change CtrlxMK-801	Fold change CtrlxCloz	Fold change CtrlxMK-801/Cloz	Location	Biological process
AKAP10	A kinase (PRKA) anchor protein 10	1.77	> -5	> -5	Cytoplasm	Cell communication and signaling
GDI2	Rab GDP dissociation inhibitor beta	>5	-4.76	>5	Cytoplasm	Cell communication and signaling
PHB	Prohibitin	>5	1.56	>5	Nucleus	Cell communication and signaling
LMNB1	Lamin B1	2.65	-4.35	1.71	Nucleus	Cell growth and maintenance
ALDOC	Aldolase C. fructose-bisphosphate	>5	-3.45	2.24	Cytoplasm	Energy metabolism
PRDX6	Peroxiredoxin 6	3.53	3.78	>5	Cytoplasm	Energy metabolism
CCT7	Chaperonin containing TCP1. Subunit 7 (eta)	2.28	3.96	1.58	Cytoplasm	Protein metabolism
DNAJB11	DnaJ (Hsp40) homolog. Subfamily B. Member 11	-4.17	-2.50	-3.33	Cytoplasm	Protein metabolism
EEF2	Eukaryotic translation elongation factor 2	>5	> -5	>5	Cytoplasm	Protein metabolism
NPM1	Nucleophosmin (nucleolar phosphoprotein B23. numatrin)	>5	-3.03	>5	Nucleus	Protein metabolism
PDIA3	Protein disulfide isomerase family A. member 3	3.00	-4.54	-3.45	Cytoplasm	Protein metabolism
RPL17	Ribosomal protein L17	-3.03	-3.85	-2.13	Cytoplasm	Protein metabolism
RPS16	Ribosomal protein S16	>5	3.59	>5	Cytoplasm	Protein metabolism
ANP32B	Acidic leucine-rich nuclear phosphoprotein 32 family member B	-2.22	-2.86	-2.13	Nucleus	Regulation of nucleic acid metabolism
RALY	RNA-binding protein Raly	>5	-2.44	>5	Nucleus	Regulation of nucleic acid metabolism
RBMX	RNA-binding motif protein, X chromosome	-2.63	2.41	-2.222	Nucleus	Regulation of nucleic acid metabolism


		Upregulation		Downregulation		


### MK-801-Treated Oligodendrocytes with Added Clozapine

The addition of clozapine after 4 h to the MK-801-treated cells affected the expression levels of 45 proteins, involved in six different biological processes. Most of these proteins are associated with energy metabolism, oxidative stress, and protein metabolism, and included changes in ALDOA, peroxiredoxin-6 (PRDX6), NPM1 and RPS16. In addition, profilin-1 (PFN1), a protein associated with cell growth and maintenance, showed the highest fold change of the proteins in this group (**Supplementary Table S2**).

### Clozapine-Treated Oligodendrocytes

Treatment with clozapine alone induced alterations in the levels of 122 proteins in cultured oligodendrocytes. Clozapine treatment mostly induced changes in proteins associated with regulation of nucleic acid metabolism, such as histone H2B (HIST1H2BJ), polyadenylate binding protein (PABPC1), and ribonucleoprotein (SNRPA1). In addition, ribosomal proteins as L32 (RPL32) and L8 (RPL8) were also affected (**Supplementary Table S3**). These effects are likely to represent early changes in the transcriptional and translational machinery as part of the clozapine response in oligodendrocyte cells.

### *In silico* Systems Biology

**FIGURE 4:**
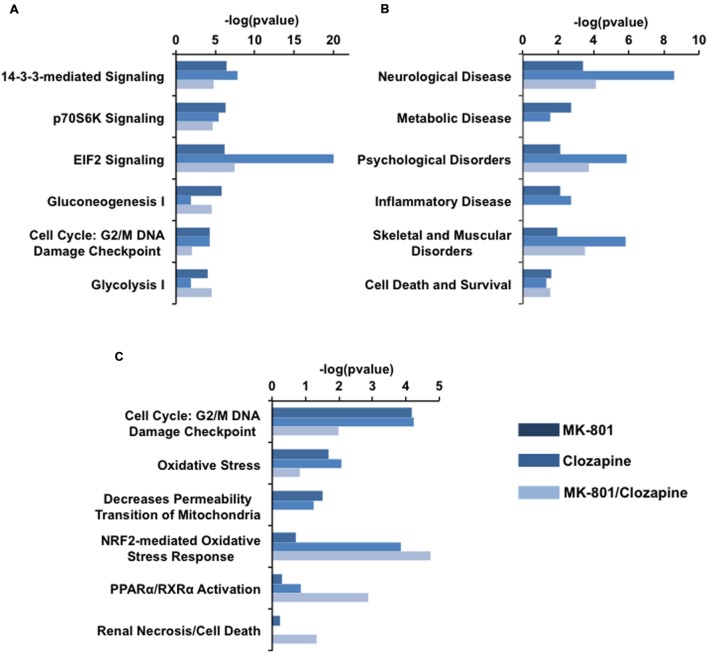
**IPA analysis of proteomic data arisen each drug treatment.** **(A)** Canonical pathways, **(B)** related disorders and Biofunctions, and **(C)** toxicity pathways generated from IPA analysis. Bars in dark blue, blue, and light blue depict MK-801, Clozapine and MK-801/Clozapine treatments, respectively.

*In silico* analysis was performed using IPA, inputting the accession codes of the differentially proteins modulated in each treatment group. The results are shown in **Figure 4**. Essentially, the obtained proteomes were associated with some canonical pathways, biofunctions, disorders, and toxicities.

Among the canonical pathways, proteins altered in the MK-801-treated cells were related to cell signaling pathways mediated by 14-3-3 protein kinase, p70S6K and by the factor eIF2 at approximately equal levels. In contrast, the proteins altered by clozapine treatment showed a marked relationship to eIF2 signaling, followed by 14-3-3-mediated and p7056k signaling. The proteome changes in MK-801-treated oligodendrocytes with the addition of clozapine showed effects similar to the MK-801 treatment alone with additional changes in proteins involved in carbohydrate metabolism pathways (**Figure 4A**).

The MK-801 treatment resulted in similar values for all groups in the area of biofunctions and disorders, except for neurological disorder group, which showed the highest score. However, the altered proteins in the clozapine-treated cells were linked with neurological disease as well as psychological, and skeletal and muscular disorders (**Figure 4B**).

Toxicity pathways generated by the analysis were associated mainly with NRF2 oxidative stress and cell cycle G2/M DNA damage responses in the clozapine treatment group (**Figure 4C**). Furthermore, the proteome changes associated with the MK-801 treatment were mainly linked to reduction of mitochondrial membrane permeability, oxidative stress, and cell cycle G2/M DNA damage responses.

**FIGURE 5:**
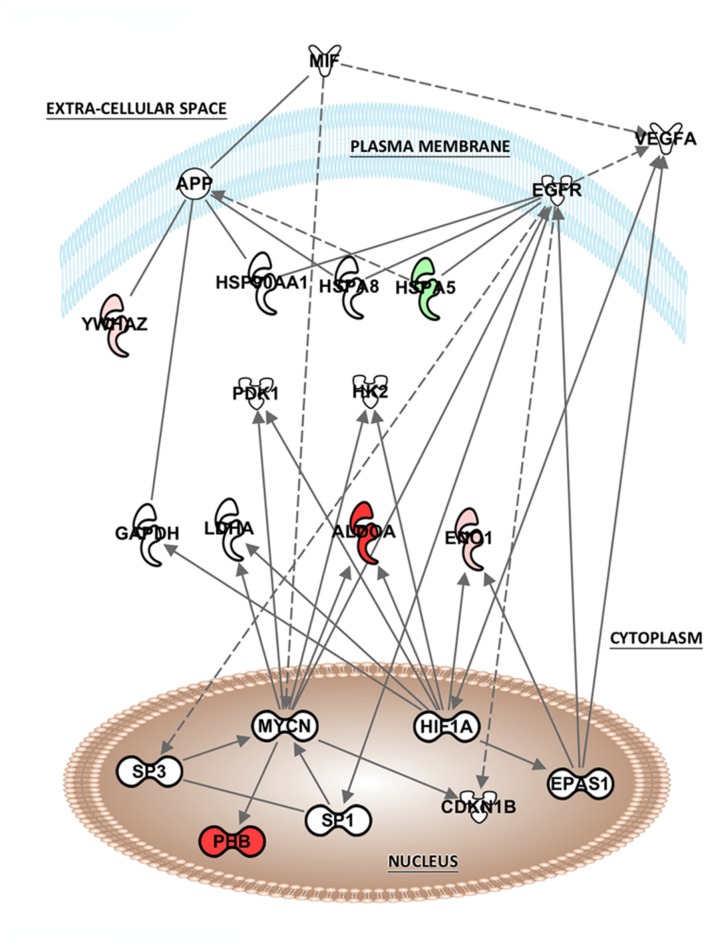
**Potential network interactions, and their interactors, of differentially expressed proteins of MK-801-treated oligodendrocytes.** The network was generated from differentially expressed proteins by IPA (see “Pathway and Functional Correlation Analysis”). Colored interactors represent proteins previous found in the proteome. Full and dashed lines depict direct and indirect connections respectively.

The list of altered proteins from the MK-801 treatment was also analyzed in terms of protein networks generated by the IPA software. This analysis can identify binding or interacting proteins which have not been detected by the proteomic approach (**Figure 5**). Hypoxia-inducing factor 1-α (HIF1-α), endothelial PAS domain-containing protein 1 (EPAS1), and MYC-proto-oncogene protein (MYCN), were the most associated transcription factors. In addition, proteins involved in energy metabolism, kinases, and heat shock proteins (HSPs) were the highly represented in the network generated (**Figure 5**).

## Discussion

One of major challenges in neuropsychiatric research is the relatively limited knowledge of the molecular mechanisms of these diseases. The development of new preclinical models to improve such knowledge is also challenging for the same reasons. Biological assays using animals or cellular cultures as models are still emerging, with the aim to provide more information about the acute effects caused by administration of neuromodulators such as MK-801 and antipsychotic drugs (Paulson et al., 2004, 2007; Ji et al., 2009a,b; Ma et al., 2009; Martins-de-Souza et al., 2011b; Ahmed et al., 2012; Palmowski et al., 2014). Considering the known effects of oligodendrocyte dysfunction in schizophrenia, we have investigated the acute response of oligodendrocytic cells to the NMDA receptor antagonist MK-801 and clozapine treatment, by identification of protein abundance changes using quantitative mass-spectrometry based proteomics. Analysis of quantitative data allowed the identification of proteins linked to a series of cellular processes and functions affected by MK-801 and showed how clozapine may block the effects of MK-801 in oligodendrocytic cells.

### Biological Process

Taken together, the different treatment groups resulted in changes in the levels of more than 200 proteins. Further studies are warranted to determine whether or not those proteins modulated by the MK-801 treatment are linked in any way to schizophrenia. This would help to establish the validity of MK-801-treated oligodendrocytes as a potential model of some aspects of schizophrenia. On the other hand, some of the proteins modulated by the clozapine treatment may be biomarkers of drug response and some could provide links to novel therapeutic targets. The combined 200 proteins were mainly associated to “cell communication and signaling,” “energy metabolism,” “cell growth and maintenance,” “protein metabolism,” and “regulation of nucleic acid metabolism” in terms of biological processes (**Figure 3**). Several of these proteins have also been reported to be differentially expressed in brain tissue samples of schizophrenia patients (Nascimento and Martins-de-Souza, 2015) and in animals treated with such drugs (Paulson et al., 2004, 2007; Ji et al., 2009a,b; Ma et al., 2009; Palmowski et al., 2014). Similarly, a previous proteomic analysis was performed to understand the effects of MK-801 on cultured astrocytes (Martins-de-Souza et al., 2011b).

In the present study, proteins associated to energy metabolism were mostly upregulated in the MK-801 treated oligodendrocyte cell line (**Supplementary Table S2**). This effect is consistent with a number of studies which have used NMDAr antagonist treatment to investigate its effects on neuronal cell cultures (Guest et al., 2015) and rat brains (Paulson et al., 2004, 2007; Zhou et al., 2012). Similar effects have been also been reported in proteomic and transcriptomic studies using post-mortem samples from schizophrenia patients (Martins-de-Souza, 2010; Martins-de-Souza et al., 2011a; Cassoli et al., 2015). Taken together, these findings suggest that dysfunctions of NMDA activity can cause disruptions in energy metabolism pathways and vice-versa. However, further studies are necessary to extrapolate these findings to schizophrenia pathophysiology.

Effects of clozapine on cell cultures have already been reported. This drug induced oxidation of proteins involved in energy metabolism in SKNSH neuroblastoma cells, seen as effects on mitochondrial ribosomal protein S22 (MRPS22), mitochondrial malate dehydrogenase (MDH), calumenin (CALU), pyruvate kinase (PK1), and 3-oxoacid CoA transferase (OXCT1; Walss-Bass et al., 2008). Likewise, this antipsychotic provoked an increased oxidation of specific proteins, such as enolase (ENO), triosephosphate isomerase (TPI), glyceraldehyde-3-phosphate dehydrogenase (GAPD), Rho GDP dissociation inhibitor (GDI), cofilin (CFL), uridine monophosphate/cytidine monophosphate (UMP-CMP) kinase, and translation elongation factor, in lymphoblastoid cells obtained from patients with schizophrenia compared to those from healthy subjects (Baig et al., 2010). In this study, we also found that clozapine influenced the expression levels of proteins from the same biological processes in oligodendrocyte cells.

### Effects of Clozapine on MK-801-Treated Cells

**FIGURE 6:**
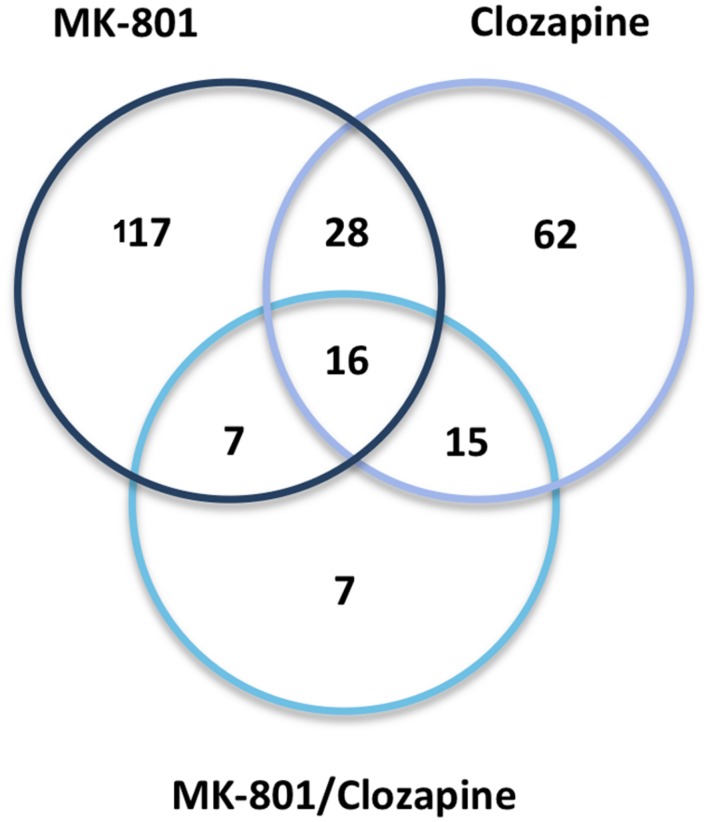
**Venn diagram.** Protein overlap between the three drug treatments (MK-801, Clozapine, and MK-801, and Clozapine) applied to the characterization of oligodendrocyte proteomes.

Treatment with clozapine appeared to reverse some of the proteome changes caused by the MK-801 treatment, suggesting that such proteins might be associated with the medication response. Additionally, 16 proteins were affected in a similar manner in all three treatment groups (**Table 1**; **Figure 6**). Some of these proteins showed opposite directional changes depending on whether cells were treated with MK-801, clozapine or both. This suggests that these proteins may be involved in both the pathophysiology and the medication response. Such proteins included the AKAP10, ALDOC, protein disulfide isomerase family A member 3 (PDIA3), PHB, Rab GDP dissociation inhibitor beta (GDI2), and PRDX6 (**Table 1**), which are discussed below.

AKAP10 levels were increased by the MK-801 treatment and decreased by clozapine. The AKAP family of scaffold proteins are a diverse group of functionally related proteins that anchor the cAMP-dependent protein kinase (PKA) as well as other signaling proteins to coordinate signal transduction in different subcellular locations (Sanderson and Dell’Acqua, 2011). They have also been associated with synaptic plasticity, which is induced by activation of NMDA-type glutamate receptors. This suggests that clozapine may act on processes related to synaptic plasticity via effects on AKAP10. Moreover, this protein has been found to be differentially expressed in samples of corpus callosum from schizophrenia patients (Saia-Cereda et al., 2015).

Aldolase C has been previously reported to be upregulated in the frontal cortex (FC), insulate cortex (IC) and dorsolateral prefrontal cortex (DLPFC) and downregulated in the prefrontal cortex (PFC), Wernicke’s area (WA), anterior temporal lobe (ATL) and anterior cingulate cortex (ACC) of schizophrenia patients compared to controls (Martins-de-Souza et al., 2011a). ALDOC is a glycolytic enzyme that catalyzes the reversible aldol cleavage of fructose-1,6-biphosphate and fructose 1-phosphate to produce dihydroxyacetone phosphate and either glyceraldehyde-3-phosphate or glyceraldehyde, respectively. In the present study, we found that the clozapine co-treatment resulted in a lowering of ALDOC levels in relation to increased levels induced by the MK-801 treatment. Disturbances in ALDOC expression, and expression of the ALDOA isoform have also been reported by studies involving MK-801-treated animals and astrocyte cultures (Paulson et al., 2004; Martins-de-Souza et al., 2011b).

Clozapine treatment resulted in increased levels of PDIA3, a protein belonging to a family of enzymes that introduces disulfide bonds into proteins and catalyzes rearrangement of incorrect disulfide bonds (Wilkinson and Gilbert, 2004). Distinct from the present result, protein previous study showed that PDIA3 was downregulated after treatment of astrocytes with MK-801 (Martins-de-Souza et al., 2011b). This suggests that MK-801 and clozapine may have opposite effects on PDIA3 levels in oligodendrocytes and astrocytes. The MK-801 treatment promoted an upregulation of PHB in oligodendrocytes, which was similarly upregulated in the DLPFC and ACC, and decreased in the PFC of schizophrenia patient brain samples (Smalla et al., 2008; Martins-de-Souza et al., 2010). PHB is a multifunctional protein which acts as a chaperone for respiration chain proteins, a general structuring scaffold in mitochondrial morphology, and it is involved in cell proliferation and transcriptional modulation (Zhou and Qin, 2013; Peng et al., 2015). Rats treated with ketamine – another NMDAr antagonist – have also shown PHB upregulation in post-synaptic density preparations of DLPFC from chronic schizophrenia patients (Smalla et al., 2008). Oligodendrocytes isolated from the DLPFC have also been found to have increased levels of PHB (Bernstein et al., 2012). Likewise, a recent proteomic study showed PHB upregulation in the peripheral blood serum and brain tissue from rats acutely treated with ketamine (Wesseling et al., 2015). Taken together, these findings indicate a relationship of PHB increased expression and hypofunction of NMDA receptor signaling.

Although, the Rab GDP dissociation inhibitor beta (GDI2) has not been found to be altered in schizophrenia proteomic studies, the Rab GDP dissociation inhibitor alpha (GDI1) isoform was previously found to be upregulated in samples of ACC and DLPFC of schizophrenia patients (English et al., 2009; Martins-de-Souza et al., 2010). The Rab proteins belong to the GDP dissociation inhibitor protein family, which regulates the GDP-GTP exchange reaction of members of small GTP-binding proteins. They are mainly involved in vesicular trafficking of molecules between cellular organelles (Shisheva et al., 1994). In our results, GDI2 was oppositely regulated by MK-801 and clozapine. However, addition of the antipsychotic to the MK-801-treated cultures was not sufficient to completely block GDI2 upregulation.

Another protein modulated by both MK-801 and clozapine in oligodendrocytes was peroxiredoxin 6 (PRDX6). The peroxiredoxins are a family of antioxidant enzymes that are ubiquitously distributed in the cell and can control cytokine-induced peroxide levels, which mediate signal transduction in mammalian cells. High abundance of peroxiredoxins in mammalian cells appears to protect the cellular components by removing accumulated peroxides produced as a result of normal cellular metabolism or as a response to oxidative stress (Rhee et al., 2005). In our study, PRDX6 was found to be upregulated by MK-801 and the clozapine single treatments. Interestingly, this effect was enhanced by the co-treatment resulting in markedly higher levels of this protein. PRDX6 has also been found to be altered in DLPFC and WA from schizophrenia patients (Martins-de-Souza et al., 2009a,b) as well as in MK-801-treated astrocytes (Martins-de-Souza et al., 2011b). An upregulation of PRDX6 following haloperidol treatment in rats has also been described (Andreazza et al., 2015), although the same study suggested that clozapine may also induce oxidative stress in liver, consistent with the documented adverse effects of this drug. Taken together, these findings show a potential connection between neurotransmission dysfunctions and oxidative damage, which might be relevant to disease pathogenesis (Ben-Shachar and Laifenfeld, 2004; Rajasekaran et al., 2015). Furthermore, the phospholipase A2 (PLA2) activity of PRDX6 is critical for regulation of phospholipid turnover (Chen et al., 2000) and differential regulation of PRDX6 may accelerate phospholipid turnover. Both of these PRDX6 activities may be related to schizophrenia pathogenesis since an enhancement of phospholipid turnover has been previously reported in schizophrenia frontal lobe (Gattaz et al., 1990). Therefore, PRDX6 could function as a brain marker for schizophrenia.

### *In silico* Functional Correlations

Functional correlation of the regulated proteins revealed that MK-801 and clozapine treatment could lead to alterations in the canonical signaling pathways mediated by 14-3-3 protein kinase, p70S6K and eIF2 factor. In addition, changes in carbohydrate metabolism pathways were also detected.

The 14-3-3 proteins compose a family of highly conserved acidic proteins, with molecular weights of 25–30 kD. There are seven mammalian 14-3-3 isoforms in eukaryotic cells which function as adaptor or “chaperone molecules” and scaffolding proteins that can translocate freely from the cytoplasm to the nucleus and vise-versa (Muslin et al., 1996). They are found as homo- or heterodimers and interact with cellular proteins representing a wide range of processes, such as neuronal development, mitogenic signal transduction, apoptotic cell death, cell cycle and cell growth controls (Mhawech, 2005). Several reports have shown disturbances in the expression of this protein family in schizophrenia brain tissue (Hayakawa et al., 1998; Wong et al., 2003; Middleton et al., 2005; Martins-de-Souza et al., 2012; Saia-Cereda et al., 2015; Schubert et al., 2015). Moreover, schizophrenia-related behavioral phenotypes have been described in 14-3-3 functional knockout mice (Cheah et al., 2012; Foote et al., 2015). Here, MK-801 and clozapine treatments resulted in increased levels of 14-3-3 proteins. Interestingly, when clozapine was administered to MK-801-treated cells, the levels of 14-3-3 protein theta (14-3-3ζ) decreased and signaling mediated by the 14-3-3 family appeared to be abolished. Other studies have described changes in this protein family in the PFC of subjects with schizophrenia as a normalizing effect of antipsychotics, such as clozapine (Rivero et al., 2015). Furthermore, clozapine has been used to rescue the locomotor hyperactivity of 14-3-3ζ knockout mice, which may indicate a novel role for 14-3-3ζ in dopaminergic neurotransmission (Ramshaw et al., 2013). Considering these findings, further studies should be performed to determine whether or not dysregulation of 14-3-3 expression in oligodendrocytes is involved in schizophrenia, as suggested in by some post-mortem studies.

A previous study reported a decrease in phosphorylation of p70S6K and its substrates in frontal cortices of 7-days-old rats that were acutely treated with MK-801 (Yoon et al., 2008). Although, MK-801 is an NMDAr antagonist, the present results indicated that it may also antagonize p70S6K signaling. In contrast, the clozapine and co-MK-801/clozapine co-treatment led to increased expression of proteins involved in p70S6K signaling. The p70S6K protein is a serine/threonine kinase that phosphorylates the ribosomal S6 subunit, a component of the 40S subunit of eukaryotic ribosomes. It is activated by mTOR in mitogenic pathways downstream of phosphoinositide 3 kinase (PI3K) and plays a role in protein synthesis and in cell growth control (Fenton and Gout, 2011). Thus, the observed upregulation of the p70S6K pathway promoted by MK-801 and clozapine in oligodendrocytes might represent an adaptive response resulting in increased translation initiation in protein synthesis.

The initiation phase of protein synthesis, during which ribosomes select mRNAs to be translated, and identify the translational start site, requires a set of EIFs (eukaryotic translation initiation factors). EIF2 (eukaryotic initiation factor-2) is a GTP (guanosine triphosphate)-binding protein that enables transport of the initiation-specific form of Met-tRNA (Met-tRNAi) onto the ribosome. According to IPA analysis, all drug treatments affected the expression of many proteins related to EIF2 signaling in oligodendrocytes. This effect seems to activate the pathway and may reflect the increased protein synthesis in the cells. Members of EIFs have been found functionally linked to the disrupted in schizophrenia 1 protein (DISC1) and to stress granules (Ogawa et al., 2005). Moreover, many risk-promoting genes and a number of environmental risk factors are related to oligodendrocyte cell loss and hypomyelination via activation of EIF2-alpha kinases in schizophrenia (Carter, 2007). These can lead to an arrest of protein synthesis through the eventual inhibition of translation initiation factor EIF2-beta by phosphorylated eIF2-alpha (Carter, 2007).

### Toxicity Pathways

The toxicity pathways associated with the proteomic changes of all treatment groups were mainly cell cycle G2/M DNA damage and nuclear factor-erythroid 2-related factor 2 (NRF2)-mediated responses. The link to cell cycle G2/M DNA damage signaling was due to changes in the 14-3-3 family proteins (YWHAB, YWHAG, YWHAH, YWHAQ, and YWHAZ), in addition to the Wee1-like protein kinase (WEE1).

Other proteins predicted to be involved in drug treatment responses included alcohol dehydrogenase [NADP(+)] (AKR1A1), T-complex protein 1 subunit eta (CCT7), DnaJ homolog subfamily B member 11 (DNAJB11), dual specificity mitogen-activated protein kinase kinases 1 (MAP2K1) and 2 (MAP2K2), although this occurred most predominantly in the MK-801 and clozapine co-treatment condition. NRF2 is a reactive oxygen species-responsive transcription factor, which binds to the antioxidant response elements (AREs) within the promoter of antioxidant enzyme genes and activates their transcription. NRF2 signaling takes place upon exposure of cells to oxidative stress through phosphorylation of the protein in response to activation of protein kinase C, phosphatidylinositol 3-kinase and MAP kinase pathways (Petri et al., 2012). After phosphorylation, NRF2 translocates to the nucleus, binds AREs and transactivates detoxifying enzymes and antioxidant enzymes, such as glutathione *S*-transferase, NAD(P)H quinone oxidoreductase, sulfiredoxin 1 (SRXN1) and thioredoxin reductase 1 (TXNRD1). SRXN1 and TXNRD1 are involved in the reduction and recovery of peroxiredoxins (Neumann et al., 2009; Soriano et al., 2009). The current results suggest that differential expression of NRF2 signaling proteins can affect PRDXs expression, although additional analyses should be performed to confirm these findings.

### Potential Interactions

The analysis of potential protein interactions showed that MK-801 treatment of oligodendrocytes included changes in proteins linked to other proteins which were mostly associated with energy and protein metabolism. However, nuclear transcription factors also appeared as highly connected proteins such as hypoxia-inducing factor 1-α (HIF1-α), MYCN and endothelial PAS domain-containing protein 1 (EPAS1). This suggests that these factors may be upstream regulators of the energy metabolism proteins altered by the MK-801 treatment.

HIF1-α is a subunit of a heterodimeric transcription factor hypoxia-inducible factor 1 (HIF-1) encoded by the *HIF1A* gene. HIF1 is the main transcriptional factor regulating the effects of hypoxia in expression of genes for angiogenesis, glycolysis and protective responses (Semenza, 2001). This factor is important in embryonic development and neural fold formation (Maltepe and Simon, 1998). Moreover, HIF1 has been related to regulation of many risk genes of schizophrenia. A meta-analysis study revealed that more than 50% of schizophrenia candidate genes met the criteria for a link to ischemia, hypoxia and/or vascular factors (Schmidt-Kastner et al., 2012) and epidemiological studies have shown that, events that lead to development of fetal hypoxia and inflammation during pregnancy are associated with increased risk of schizophrenia later in life (Nicodemus et al., 2008; Khandaker et al., 2013).

### Final Remarks

Considering all findings discussed above, the current study supports the concept that dysfunctions in NMDAr signaling in oligoendrocytes may be a central process in schizophrenia. MK-801-treated oligodendrocytes exhibited differences on cellular processes that have been previously observed in schizophrenia samples and have also revealed new pathways that could be pivotal in the development of this disorder. Although, the MK-801 treated oligodendrocyte model cannot reflect the pathophysiology of a complex psychiatric disorder as schizophrenia in its entirety, employing this approach might provide insights about specific aspects of the disease and lead to a novel preclinical tool for drug target discovery

## Author Contributions

JC helped in experimental design, analyzed and interpreted the data and wrote the original version of the manuscript. Also corrected the manuscript according to reviewers advice. KI performed cell cultures experiments and pharmacological treatments. Helped in data interpretation and manuscript revision. JS has interpreted the data and has revised the manuscript carefully. PG analyzed the data and rewrote the manuscript carefully. CT analyzed the data and has revised the manuscript carefully. JN analyzed and interpreted the data and revised the manuscript carefully. DM-d-S conceptualized the study and experimental design. Analyzed the data and revised critically the original version of the manuscript. Later, corrected the manuscript according to reviewers advice.

## Conflict of Interest Statement

The authors declare that the research was conducted in the absence of any commercial or financial relationships that could be construed as a potential conflict of interest.

## Footnotes

1 http://www.hprd.org/

2 www.qiagen.com/ingenuity
