# Supplementary material for: Effect of MK-801 and Clozapine on the Proteome of Cultured Human Oligodendrocytes
Source: Front Cell Neurosci. 2016 Mar 3;10:52. doi: 10.3389/fncel.2016.00052 (PMC4776125; doi:10.3389/fncel.2016.00052)
Supplement: Supplementary file 1 [file Table_1.DOCX]

Supplementary Material

**Schizophrenia from an *in vitro* perspective: the role of glutamatergic transmission and oligodendrocytes employing MK-801 and clozapine**

Juliana S. Cassoli^1^, Keiko Iwata^2^, Johann Steiner^3^, Paul C. Guest^1^, Christoph W. Turck^4^, Juliana M. Nascimento^1,5^, Daniel Martins-de-Souza^1,6^*

*** Correspondence:** Corresponding Author: dmsouza@unicamp.br

**Supplementary Table 1. Differentially expressed proteins from oligodendrocytes (MO3.13 cells) after acute treatment with 50mM MK-801 classified by their biological and molecular functions.**

| **Acession** | **Gene name** | **Protein name** | **Score** | **Mass (Da)** | **Ctrl x MK801** | **Regulation** | **Nº Peptides** | **Bio. Process** | **Mol. Function** |
| --- | --- | --- | --- | --- | --- | --- | --- | --- | --- |
| I5P1_HUMAN | INPP5A | Type I inositol 1,4,5-trisphosphate 5-phosphatase | 64 | 48359 | -9.09 | 🡻 | 4 | Cell communication & Signalling | Lipid phosphatase activity |
| NDKB_HUMAN | NME2 | Nucleoside diphosphate kinase B | 641 | 17401 | -2.94 | 🡻 | 11 | Cell communication & Signalling | Transcription factor activity |
| RB11A_HUMAN | RAB11A | Ras-related protein Rab-11A | 393 | 24492 | 2.14 | 🡹 | 4 | Cell communication & Signalling | GTPase activity |
| ANXA6_HUMAN | ANXA6 | Annexin A6 | 733 | 76168 | 2.50 | 🡹 | 5 | Cell communication & Signalling | Calcium ion binding |
| RAN_HUMAN | RAN | GTP-binding nuclear protein Ran | 619 | 24579 | 6.46 | 🡹 | 9 | Cell communication & Signalling | GTPase activity |
| ANXA5_HUMAN | ANXA5 | Annexin A5 | 888 | 35971 | 10 | 🡹 | 3 | Cell communication & Signalling | Calcium ion binding |
| F10A1_HUMAN | ST13 | Hsc70-interacting protein | 66 | 41477 | 1.57 | 🡹 | 5 | Cell communication & Signalling | Receptor signaling complex scaffold activity |
| PHB_HUMAN | PHB | Prohibitin | 2673 | 29843 | 9.49 | 🡹 | 14 | Cell communication & Signalling | Receptor signaling complex scaffold activity |
| 1433B_HUMAN | YWHAB | 14-3-3 protein beta/alpha | 807 | 28179 | 1.70 | 🡹 | 14 | Cell communication & Signalling | Receptor signaling complex scaffold activity |
| 1433F_HUMAN | YWHAH | 14-3-3 protein eta | 572 | 28372 | 1.72 | 🡹 | 6 | Cell communication & Signalling | Receptor signaling complex scaffold activity |
| 1433G_HUMAN | YWHAG | 14-3-3 protein gamma | 940 | 28456 | 1.72 | 🡹 | 10 | Cell communication & Signalling | Receptor signaling complex scaffold activity |
| AKA10_HUMAN | AKAP10 | A-kinase anchor protein 10, mitochondrial | 107 | 74228 | 1.77 | 🡹 | 8 | Cell communication & Signalling | Cytoskeletal anchoring activity |
| ACTN4_HUMAN | ACTN4 | Alpha-actinin-4 | 1959 | 105245 | -2.94 | 🡻 | 7 | Cell growth & maintenance | Structural constituent of cytoskeleton |
| ACTN1_HUMAN | ACTN1 | Alpha-actinin-1 | 1593 | 103563 | -2.70 | 🡻 | 5 | Cell growth & maintenance | Cytoskeletal protein binding |
| TBB5_HUMAN | TUBB | Tubulin beta chain | 3418 | 50095 | -2.56 | 🡻 | 7 | Cell growth & maintenance | Structural constituent of cytoskeleton |
| TBB2C_HUMAN | TUBB4B | Tubulin beta-4B chain | 3044 | 50255 | -2.13 | 🡻 | 4 | Cell growth & maintenance | Structural molecule activity |
| TBB3_HUMAN | TUBB3 | Tubulin beta-3 chain | 2189 | 50856 | -2.13 | 🡻 | 3 | Cell growth & maintenance | Structural molecule activity |
| 1433Z_HUMAN | YWHAZ | 14-3-3 protein zeta/delta | 1266 | 27899 | 1.72 | 🡹 | 10 | Cell growth & maintenance | Receptor signaling complex scaffold activity |
| FSCN1_HUMAN | FSCN1 | Fascin | 2702 | 55123 | 1.87 | 🡹 | 5 | Cell growth & maintenance | Structural molecule activity |
| LMNB1_HUMAN | LMNB1 | Lamin-B1 | 1104 | 66653 | 2.65 | 🡹 | 5 | Cell growth & maintenance | Structural molecule activity |
| GANAB_HUMAN | GANAB | Neutral alpha-glucosidase AB | 1288 | 107263 | -2.44 | 🡻 | 6 | Energy Metabolism | Hydrolase activity |
| PRDX4_HUMAN | PRDX4 | Peroxiredoxin-4 | 1034 | 30749 | -1.64 | 🡻 | 8 | Energy Metabolism | Peroxidase activity |
| ENOA_HUMAN | ENO1 | Alpha-enolase | 3002 | 47481 | 2.29 | 🡹 | 14 | Energy Metabolism | Catalytic activity |
| PRDX6_HUMAN | PRDX6 | Peroxiredoxin-6 | 1502 | 25133 | 3.53 | 🡹 | 6 | Energy Metabolism | Peroxidase activity |
| TKT_HUMAN | TKT | Transketolase | 542 | 68519 | 4.35 | 🡹 | 4 | Energy Metabolism | Transferase activity, transferring aldehyde or ketonic groups |
| ALDOA_HUMAN | ALDOA | Fructose-bisphosphate aldolase A | 1586 | 39851 | 10 | 🡹 | 7 | Energy Metabolism | Lyase activity |
| ALDOC_HUMAN | ALDOC | Fructose-bisphosphate aldolase C | 650 | 39830 | 10 | 🡹 | 3 | Energy Metabolism | Lyase activity |
| MDHM_HUMAN | MDH2 | Malate dehydrogenase, mitochondrial | 868 | 35937 | 10 | 🡹 | 7 | Energy Metabolism | Catalytic activity |
| AN32A_HUMAN | ANP32A | Acidic leucine-rich nuclear phosphoprotein 32 family member A | 812 | 28682 | -1.56 | 🡻 | 8 | Immune response | MHC class I receptor activity |
| DJB11_HUMAN | DNAJB11 | DnaJ homolog subfamily B member 11 | 60 | 40774 | -4.17 | 🡻 | 3 | Protein metabolism | Chaperone activity |
| GRP78_HUMAN | HSPA5 | 78 kDa glucose-regulated protein | 3761 | 72402 | -3.33 | 🡻 | 11 | Protein metabolism | Chaperone activity |
| RL17_HUMAN | RPL17 | 60S ribosomal protein L17 | 149 | 21611 | -3.03 | 🡻 | 4 | Protein metabolism | Structural constituent of ribosome |
| PSB4_HUMAN | PSMB4 | Proteasome subunit beta type-4 | 284 | 29243 | -2.63 | 🡻 | 4 | Protein metabolism | Ubiquitin-specific protease activity |
| IF4A1_HUMAN | EIF4A1 | Eukaryotic initiation factor 4A-I | 1612 | 46353 | 1.56 | 🡹 | 11 | Protein metabolism | Translation regulator activity |
| RS4X_HUMAN | RPS4X | 40S ribosomal protein S4, X isoform | 1021 | 29807 | 1.61 | 🡹 | 7 | Protein metabolism | Structural constituent of ribosome |
| RS6_HUMAN | RPS6 | 40S ribosomal protein S6 | 148 | 28834 | 1.71 | 🡹 | 7 | Protein metabolism | Structural constituent of ribosome |
| RL9_HUMAN | RPL9 | 60S ribosomal protein L9 | 298 | 21964 | 1.73 | 🡹 | 6 | Protein metabolism | Structural constituent of ribosome |
| TBCB_HUMAN | TBCB | Tubulin-folding cofactor B | 745 | 27594 | 2.04 | 🡹 | 4 | Protein metabolism | Chaperone activity |
| IF4H_HUMAN | EIF4H | Eukaryotic translation initiation factor 4H | 794 | 27425 | 2.25 | 🡹 | 4 | Protein metabolism | Translation regulator activity |
| TCPH_HUMAN | CCT7 | T-complex protein 1 subunit eta | 745 | 59842 | 2.28 | 🡹 | 5 | Protein metabolism | Chaperone activity |
| CYBP_HUMAN | CACYBP | Calcyclin-binding protein | 197 | 26308 | 2.37 | 🡹 | 4 | Protein metabolism | Molecular function unknown |
| RS2_HUMAN | RPS2 | 40S ribosomal protein S2 | 159 | 31590 | 2.61 | 🡹 | 4 | Protein metabolism | Structural constituent of ribosome |
| PDIA3_HUMAN | PDIA3 | Protein disulfide-isomerase A3 | 1696 | 57146 | 3.00 | 🡹 | 8 | Protein metabolism | Isomerase activity |
| EF2_HUMAN | EEF2 | Elongation factor 2 | 3461 | 96246 | 5.53 | 🡹 | 16 | Protein metabolism | Translation regulator activity |
| RL7A_HUMAN | RPL7A | 60S ribosomal protein L7a | 256 | 30148 | 9.43 | 🡹 | 4 | Protein metabolism | Structural constituent of ribosome |
| RS16_HUMAN | RPS16 | 40S ribosomal protein S16 | 139 | 16549 | 10 | 🡹 | 6 | Protein metabolism | Structural constituent of ribosome |
| NPM_HUMAN | NPM1 | Nucleophosmin | 837 | 32726 | 10 | 🡹 | 5 | Protein metabolism | Chaperone activity |
| TCPG_HUMAN | CCT3 | T-complex protein 1 subunit gamma | 1617 | 61066 | 10 | 🡹 | 3 | Protein metabolism | Chaperone activity |
| FBRL_HUMAN | FBL | rRNA 2'-O-methyltransferase fibrillarin | 330 | 33877 | -2.94 | 🡻 | 4 | Reg. of nucleic acid metab | Ribonucleoprotein |
| U2AF1_HUMAN | U2AF1 | Splicing factor U2AF 35 kDa subunit | 305 | 28368 | -2.78 | 🡻 | 4 | Reg. of nucleic acid metab | RNA binding |
| HNRPG_HUMAN | RBMX | RNA-binding motif protein, X chromosome | 545 | 42306 | -2.63 | 🡻 | 7 | Reg. of nucleic acid metab | RNA binding |
| HNRPF_HUMAN | HNRNPF | Heterogeneous nuclear ribonucleoprotein F | 1418 | 45985 | -2.04 | 🡻 | 5 | Reg. of nucleic acid metab | Ribonucleoprotein |
| HNRH1_HUMAN | HNRNPH1 | Heterogeneous nuclear ribonucleoprotein H | 3000 | 49484 | -1.89 | 🡻 | 5 | Reg. of nucleic acid metab | Ribonucleoprotein |
| ROA3_HUMAN | HNRNPA3 | Heterogeneous nuclear ribonucleoprotein A3 | 1297 | 39799 | -1.82 | 🡻 | 5 | Reg. of nucleic acid metab | RNA binding |
| H2A1B_HUMAN | HIST1H2AB | Histone H2A type 1-B/E | 2418 | 14127 | -1.79 | 🡻 | 10 | Reg. of nucleic acid metab | DNA binding |
| H4_HUMAN | HIST1H4A | Histone H4 | 898 | 11360 | -1.69 | 🡻 | 7 | Reg. of nucleic acid metab | DNA binding |
| DDX17_HUMAN | DDX17 | Probable ATP-dependent RNA helicase DDX17 | 1063 | 72953 | 1.54 | 🡹 | 5 | Reg. of nucleic acid metab | ATPase activity |
| H2B1J_HUMAN | HIST1H2BJ | Histone H2B type 1-J | 468 | 13896 | 2.39 | 🡹 | 4 | Reg. of nucleic acid metab | DNA binding |
| PAIRB_HUMAN | SERBP1 | Plasminogen activator inhibitor 1 RNA-binding protein | 467 | 44995 | 3.28 | 🡹 | 9 | Reg. of nucleic acid metab | RNA binding |
| H2B3B_HUMAN | HIST3H2BB | Histone H2B type 3-B | 531 | 13900 | 3.71 | 🡹 | 5 | Reg. of nucleic acid metab | DNA binding |
| H2B1A_HUMAN | HIST1H2BA | Histone H2B type 1-A | 333 | 14159 | 3.85 | 🡹 | 3 | Reg. of nucleic acid metab | DNA binding |
| PHB2_HUMAN | PHB2 | Prohibitin-2 | 2202 | 33276 | 5.30 | 🡹 | 4 | Reg. of nucleic acid metab | Transcription regulator activity |
| FUBP2_HUMAN | KHSRP | Far upstream element-binding protein 2 | 809 | 73443 | 6.52 | 🡹 | 3 | Reg. of nucleic acid metab | Transcription regulator activity |
| KU70_HUMAN | XRCC6 | X-ray repair cross-complementing protein 6 | 230 | 70084 | 7.60 | 🡹 | 4 | Reg. of nucleic acid metab | DNA binding |
| RALY_HUMAN | RALY | RNA-binding protein Raly | 254 | 32501 | 10 | 🡹 | 3 | Reg. of nucleic acid metab | RNA binding |
| SFXN1_HUMAN | SFXN1 | Sideroflexin-1 | 813 | 35881 | -2.22 | 🡻 | 3 | Transport | Transporter activity |
| GDIB_HUMAN | GDI2 | Rab GDP dissociation inhibitor beta | 935 | 51087 | 10 | 🡹 | 4 | Transport | Auxiliary transport protein activity |
