# Supplementary material for: Effect of MK-801 and Clozapine on the Proteome of Cultured Human Oligodendrocytes
Source: Front Cell Neurosci. 2016 Mar 3;10:52. doi: 10.3389/fncel.2016.00052 (PMC4776125; doi:10.3389/fncel.2016.00052)
Supplement: Supplementary file 2 [file Table_2.DOCX]

Supplementary Material

**Schizophrenia from an *in vitro* perspective: the role of glutamatergic transmission and oligodendrocytes employing MK-801 and clozapine**

Juliana S. Cassoli^1^, Keiko Iwata^2^, Johann Steiner^3^, Paul C. Guest^1^, Christoph W. Turck^4^, Juliana M. Nascimento^1,5^, Daniel Martins-de-Souza^1,6^*

*** Correspondence:** Corresponding Author: dmsouza@unicamp.br

**Supplementary Table 2. Differentially expressed proteins from oligodendrocytes (MO3.13 cells) after acute treatment with 50 mM MK-801 and 50 mM Clozapine, classified by their biological and molecular functions.**

| **Accession** | **Gene name** | **Protein name** | **Score** | **Mass (Da)** | **Ctrl x Mk+Cloz** | **Regulation** | **Nº Peptides** | **Bio.Process** | **Mol.Function** |
| --- | --- | --- | --- | --- | --- | --- | --- | --- | --- |
| PEBP1_HUMAN | PEBP1 | Phosphatidylethanolamine-binding protein 1 | 198 | 21158 | -3.13 | 🡻 | 3 | Cell communication & Signalling | Protease inhibitor activity |
| 1433T_HUMAN | YWHAQ | 14-3-3 protein theta | 735 | 28032 | -2.27 | 🡻 | 5 | Cell communication & Signalling | Receptor signaling complex scaffold activity |
| RCN1_HUMAN | RCN1 | Reticulocalbin-1 | 451 | 38866 | -1.96 | 🡻 | 5 | Cell communication & Signalling | Calcium ion binding |
| MP2K1_HUMAN | MAP2K1 | Dual specificity mitogen-activated protein kinase kinase 1 | 347 | 43753 | 2.72 | 🡹 | 4 | Cell communication & Signalling | Protein threonine/tyrosine kinase activity |
| MP2K2_HUMAN | MAP2K2 | Dual specificity mitogen-activated protein kinase kinase 2 | 190 | 44681 | 2.72 | 🡹 | 4 | Cell communication & Signalling | Protein threonine/tyrosine kinase activity |
| RAN_HUMAN | RAN | GTP-binding nuclear protein Ran | 619 | 24579 | 2.92 | 🡹 | 9 | Cell communication & Signalling | GTPase activity |
| ANXA5_HUMAN | ANXA5 | Annexin A5 | 888 | 35971 | 4.19 | 🡹 | 3 | Cell communication & Signalling | Calcium ion binding |
| PHB_HUMAN | PHB | Prohibitin | 2673 | 29843 | 5.56 | 🡹 | 14 | Cell communication & Signalling | Receptor signaling complex scaffold activity |
| AKA10_HUMAN | AKAP10 | A-kinase anchor protein 10, mitochondrial | 107 | 74228 | -11.11 | 🡻 | 7 | Cell communication & Signalling | Cytoskeletal anchoring activity |
| WEE1_HUMAN | WEE1 | Wee1-like protein kinase | 38 | 72237 | -11.11 | 🡻 | 3 | Cell communication & Signalling | Protein threonine/tyrosine kinase activity |
| LMNB1_HUMAN | LMNB1 | Lamin-B1 | 1104 | 66653 | 1.71 | 🡹 | 5 | Cell growth & maintenance | Structural molecule activity |
| TBA4A_HUMAN | TUBA4A | Tubulin alpha-4A chain | 7878 | 50634 | 1.77 | 🡹 | 15 | Cell growth & maintenance | Structural constituent of cytoskeleton |
| PROF1_HUMAN | PFN1 | Profilin-1 | 389 | 15216 | 10 | 🡹 | 3 | Cell growth & maintenance | Cytoskeletal protein binding |
| GLU2B_HUMAN | PRKCSH | Glucosidase 2 subunit beta | 455 | 60357 | -1.92 | 🡻 | 7 | Energy Metabolism | Glucosidase activity |
| ALDOC_HUMAN | ALDOC | Fructose-bisphosphate aldolase C | 650 | 39830 | 2.24 | 🡹 | 3 | Energy Metabolism | Lyase activity |
| AK1A1_HUMAN | AKR1A1 | Alcohol dehydrogenase [NADP(+)] | 229 | 36892 | 2.32 | 🡹 | 3 | Energy Metabolism | Oxidoreductase activity |
| PGK1_HUMAN | PGK1 | Phosphoglycerate kinase 1 | 861 | 44985 | 4.82 | 🡹 | 5 | Energy Metabolism | Catalytic activity |
| PRDX6_HUMAN | PRDX6 | Peroxiredoxin-6 | 1502 | 25133 | 5.82 | 🡹 | 6 | Energy Metabolism | Peroxidase activity |
| ALDOA_HUMAN | ALDOA | Fructose-bisphosphate aldolase A | 1586 | 39851 | 10 | 🡹 | 7 | Energy Metabolism | Lyase activity |
| PDIA3_HUMAN | PDIA3 | Protein disulfide-isomerase A3 | 1696 | 57146 | -3.45 | 🡻 | 8 | Protein metabolism | Isomerase activity |
| DJB11_HUMAN | DNAJB11 | DnaJ homolog subfamily B member 11 | 60 | 40774 | -3.33 | 🡻 | 3 | Protein metabolism | Chaperone activity |
| RL17_HUMAN | RPL17 | 60S ribosomal protein L17 | 149 | 21611 | -2.13 | 🡻 | 4 | Protein metabolism | Structural constituent of ribosome |
| RS18_HUMAN | RPS18 | 40S ribosomal protein S18 | 450 | 17708 | -1.85 | 🡻 | 6 | Protein metabolism | Structural constituent of ribosome |
| IF2B_HUMAN | EIF2S2 | Eukaryotic translation initiation factor 2 subunit 2 | 192 | 38707 | -1.82 | 🡻 | 6 | Protein metabolism | Translation regulator activity |
| PSA1_HUMAN | PSMA1 | Proteasome subunit alpha type-1 | 421 | 29822 | -1.79 | 🡻 | 5 | Protein metabolism | Peptidase activity |
| ENPL_HUMAN | HSP90B1 | Endoplasmin | 2334 | 92696 | -1.59 | 🡻 | 20 | Protein metabolism | Heat shock protein activity |
| RL9_HUMAN | RPL9 | 60S ribosomal protein L9 | 298 | 21964 | 1.56 | 🡹 | 6 | Protein metabolism | Structural constituent of ribosome |
| TCPH_HUMAN | CCT7 | T-complex protein 1 subunit eta | 745 | 59842 | 1.58 | 🡹 | 5 | Protein metabolism | Chaperone activity |
| PPIA_HUMAN | PPIA | Peptidyl-prolyl cis-trans isomerase A | 2202 | 18229 | 2.09 | 🡹 | 8 | Protein metabolism | Isomerase activity |
| SERPH_HUMAN | SERPINH1 | Serpin H1 | 1018 | 46525 | 2.29 | 🡹 | 4 | Protein metabolism | Heat shock protein activity |
| RS3_HUMAN | RPS3 | 40S ribosomal protein S3 | 1209 | 26842 | 4.56 | 🡹 | 6 | Protein metabolism | Structural constituent of ribosome |
| CH60_HUMAN | HSPD1 | 60 kDa heat shock protein, mitochondrial | 2786 | 61187 | 4.83 | 🡹 | 20 | Protein metabolism | Heat shock protein activity |
| EF2_HUMAN | EEF2 | Elongation factor 2 | 3461 | 96246 | 5.20 | 🡹 | 16 | Protein metabolism | Translation regulator activity |
| NPM_HUMAN | NPM1 | Nucleophosmin | 837 | 32726 | 10 | 🡹 | 5 | Protein metabolism | Chaperone activity |
| RS16_HUMAN | RPS16 | 40S ribosomal protein S16 | 139 | 16549 | 10 | 🡹 | 6 | Protein metabolism | Structural constituent of ribosome |
| ILF2_HUMAN | ILF2 | Interleukin enhancer-binding factor 2 | 449 | 43263 | -2.78 | 🡻 | 7 | Reg. of nucleic acid metab | Transcription factor activity |
| HNRPG_HUMAN | RBMX | RNA-binding motif protein, X chromosome | 545 | 42306 | -2.22 | 🡻 | 7 | Reg. of nucleic acid metab | RNA binding |
| NP1L1_HUMAN | NAP1L1 | Nucleosome assembly protein 1-like 1 | 517 | 45631 | -2.04 | 🡻 | 4 | Reg. of nucleic acid metab | DNA binding |
| FBRL_HUMAN | FBL | rRNA 2'-O-methyltransferase fibrillarin | 330 | 33877 | -2.00 | 🡻 | 4 | Reg. of nucleic acid metab | Ribonucleoprotein |
| H2A1B_HUMAN | HIST1H2AB | Histone H2A type 1-B/E | 2418 | 14127 | -1.59 | 🡻 | 10 | Reg. of nucleic acid metab | DNA binding |
| HNRPM_HUMAN | HNRNPM | Heterogeneous nuclear ribonucleoprotein M | 1387 | 77749 | -1.56 | 🡻 | 9 | Reg. of nucleic acid metab | Ribonucleoprotein |
| PAIRB_HUMAN | SERBP1 | Plasminogen activator inhibitor 1 RNA-binding protein | 467 | 44995 | 1.68 | 🡹 | 9 | Reg. of nucleic acid metab | RNA binding |
| RALY_HUMAN | RALY | RNA-binding protein Raly | 254 | 32501 | 7.94 | 🡹 | 3 | Reg. of nucleic acid metab | RNA binding |
| GDIB_HUMAN | GDI2 | Rab GDP dissociation inhibitor beta | 935 | 51087 | 6.54 | 🡹 | 4 | Transport | Auxiliary transport protein activity |
