# Supplementary material for: Effect of MK-801 and Clozapine on the Proteome of Cultured Human Oligodendrocytes
Source: Front Cell Neurosci. 2016 Mar 3;10:52. doi: 10.3389/fncel.2016.00052 (PMC4776125; doi:10.3389/fncel.2016.00052)
Supplement: Supplementary file 3 [file Table_3.DOCX]

Supplementary Material

**Schizophrenia from an *in vitro* perspective: the role of glutamatergic transmission and oligodendrocytes employing MK-801 and clozapine**

Juliana S. Cassoli^1^, Keiko Iwata^2^, Johann Steiner^3^, Paul C. Guest^1^, Christoph W. Turck^4^, Juliana M. Nascimento^1,5^, Daniel Martins-de-Souza^1,6^*

*** Correspondence:** Corresponding Author: dmsouza@unicamp.br

**Supplementary Table 3. Differentially expressed proteins after from oligodendrocytes (MO3.13 cells) acute treatment with 50 mM Clozapine, classified by their biological and molecular functions.**

| **Entry Name** | **Gene Name** | **Protein Name** | **Score** | **Mass (Da)** | **Ctrl x Cloz** | **Regulation** | **Nº Peptides** | **Bio.Process** | **Mol.Function** |
| --- | --- | --- | --- | --- | --- | --- | --- | --- | --- |
| AKA10_HUMAN | AKAP10 | A-kinase anchor protein 10, mitochondrial | 107 | 74228 | -11.11 | 🡻 | 8 | Cell communication & Signalling | Cytoskeletal anchoring activity |
| WEE1_HUMAN | WEE1 | Wee1-like protein kinase | 38 | 72237 | -11.11 | 🡻 | 3 | Cell communication & Signalling | Protein threonine/tyrosine kinase activity |
| 1433F_HUMAN | YWHAH | 14-3-3 protein eta | 572 | 28372 | 2.63 | 🡹 | 6 | Cell communication & Signalling | Receptor signaling complex scaffold activity |
| PEBP1_HUMAN | PEBP1 | Phosphatidylethanolamine-binding protein 1 | 198 | 21158 | 1.66 | 🡹 | 3 | Cell communication & Signalling | Protease inhibitor activity |
| EF1D_HUMAN | EEF1D | Elongation factor 1-delta | 960 | 31217 | 2.75 | 🡹 | 10 | Cell communication & Signalling | Guanyl-nucleotide exchange factor activity |
| GDIA_HUMAN | GDI1 | Rab GDP dissociation inhibitor alpha | 523 | 51177 | -9.09 | 🡻 | 3 | Cell communication & Signalling | GTPase activator activity |
| HINT1_HUMAN | HINT1 | Histidine triad nucleotide-binding protein 1 | 76 | 13907 | 5.05 | 🡹 | 4 | Cell communication & Signalling | ATPase activity |
| RSSA_HUMAN | RPSA | 40S ribosomal protein SA | 1963 | 32947 | 3.07 | 🡹 | 4 | Cell communication & Signalling | Cell adhesion molecule activity |
| ANXA1_HUMAN | ANXA1 | Annexin A1 | 898 | 38918 | -2.78 | 🡻 | 5 | Cell communication & Signalling | Calcium ion binding |
| RANG_HUMAN | RANBP1 | Ran-specific GTPase-activating protein | 142 | 23467 | -2.56 | 🡻 | 3 | Cell communication & Signalling | Transporter activity |
| MP2K1_HUMAN | MAP2K1 | Dual specificity mitogen-activated protein kinase kinase 1 | 347 | 43753 | -2.44 | 🡻 | 4 | Cell communication & Signalling | Protein threonine/tyrosine kinase activity |
| MP2K2_HUMAN | MAP2K2 | Dual specificity mitogen-activated protein kinase kinase 2 | 190 | 44681 | -2.44 | 🡻 | 4 | Cell communication & Signalling | Protein threonine/tyrosine kinase activity |
| RCN1_HUMAN | RCN1 | Reticulocalbin-1 | 451 | 38866 | -2.13 | 🡻 | 5 | Cell communication & Signalling | Calcium ion binding |
| ANXA6_HUMAN | ANXA6 | Annexin A6 | 733 | 76168 | -2.08 | 🡻 | 5 | Cell communication & Signalling | Calcium ion binding |
| PHB_HUMAN | PHB | Prohibitin | 2673 | 29843 | 1.56 | 🡹 | 14 | Cell communication & Signalling | Receptor signaling complex scaffold activity |
| 1433G_HUMAN | YWHAG | 14-3-3 protein gamma | 940 | 28456 | 2.27 | 🡹 | 10 | Cell communication & Signalling | Receptor signaling complex scaffold activity |
| 1433B_HUMAN | YWHAB | 14-3-3 protein beta/alpha | 807 | 28179 | 3.37 | 🡹 | 14 | Cell communication & Signalling | Receptor signaling complex scaffold activity |
| NDKB_HUMAN | NME2 | Nucleoside diphosphate kinase B | 641 | 17401 | 4.23 | 🡹 | 11 | Cell communication & Signalling | Transcription factor activity |
| DESM_HUMAN | DES | Desmin | 2241 | 53560 | -1.54 | 🡻 | 23 | Cell growth & maintenance | Structural constituent of cytoskeleton |
| TBA4A_HUMAN | TUBA4A | Tubulin alpha-4A chain | 7878 | 50634 | -1.54 | 🡻 | 16 | Cell growth & maintenance | Structural constituent of cytoskeleton |
| TBB3_HUMAN | TUBB3 | Tubulin beta-3 chain | 2189 | 50856 | 3.71 | 🡹 | 4 | Cell growth & maintenance | Structural molecule activity |
| PROF1_HUMAN | PFN1 | Profilin-1 | 389 | 15216 | 1.95 | 🡹 | 3 | Cell growth & maintenance | Cytoskeletal protein binding |
| ACTB_HUMAN | ACTB | Actin, cytoplasmic 1 | 7996 | 42052 | 2.01 | 🡹 | 38 | Cell growth & maintenance | Structural constituent of cytoskeleton |
| LMNB1_HUMAN | LMNB1 | Lamin-B1 | 1104 | 66653 | -4.35 | 🡻 | 5 | Cell growth & maintenance | Structural molecule activity |
| TPM2_HUMAN | TPM2 | Tropomyosin beta chain | 929 | 32945 | -4.17 | 🡻 | 8 | Cell growth & maintenance | Structural constituent of cytoskeleton |
| TPM3_HUMAN | TPM3 | Tropomyosin alpha-3 chain | 984 | 32856 | -4.17 | 🡻 | 8 | Cell growth & maintenance | Cytoskeletal protein binding |
| LMNA_HUMAN | LMNA | Prelamin-A/C | 1672 | 74380 | -3.03 | 🡻 | 5 | Cell growth & maintenance | Structural molecule activity |
| NEST_HUMAN | NES | Nestin | 2083 | 177788 | -2.50 | 🡻 | 5 | Cell growth & maintenance | Structural constituent of cytoskeleton |
| TBB5_HUMAN | TUBB | Tubulin beta chain | 3418 | 50095 | -2.50 | 🡻 | 7 | Cell growth & maintenance | Structural constituent of cytoskeleton |
| ACTN4_HUMAN | ACTN4 | Alpha-actinin-4 | 1959 | 105245 | -2.04 | 🡻 | 7 | Cell growth & maintenance | Structural constituent of cytoskeleton |
| ACTN1_HUMAN | ACTN1 | Alpha-actinin-1 | 1593 | 103563 | -2.04 | 🡻 | 5 | Cell growth & maintenance | Cytoskeletal protein binding |
| EF1A1_HUMAN | EEF1A1 | Elongation factor 1-alpha 1 | 1667 | 50451 | 1.53 | 🡹 | 20 | Cell growth & maintenance | Transcription regulator activity |
| ACTC_HUMAN | ACTC1 | Actin, alpha cardiac muscle 1 | 4745 | 42334 | 2.00 | 🡹 | 25 | Cell growth & maintenance | Structural constituent of cytoskeleton |
| 1433Z_HUMAN | YWHAZ | 14-3-3 protein zeta/delta | 1266 | 27899 | 2.27 | 🡹 | 10 | Cell growth & maintenance | Receptor signaling complex scaffold activity |
| TBB2C_HUMAN | TUBB4B | Tubulin beta-4B chain | 3044 | 50255 | 4.48 | 🡹 | 4 | Cell growth & maintenance | Structural molecule activity |
| PRDX6_HUMAN | PRDX6 | Peroxiredoxin-6 | 1502 | 25133 | 3.78 | 🡹 | 6 | Energy Metabolism | Peroxidase activity |
| PRDX4_HUMAN | PRDX4 | Peroxiredoxin-4 | 1034 | 30749 | 4.82 | 🡹 | 8 | Energy Metabolism | Peroxidase activity |
| MDHM_HUMAN | MDH2 | Malate dehydrogenase, mitochondrial | 868 | 35937 | 1.93 | 🡹 | 6 | Energy Metabolism | Catalytic activity |
| TPIS_HUMAN | TPI1 | Triosephosphate isomerase | 1328 | 26938 | 2.08 | 🡹 | 9 | Energy Metabolism | Isomerase activity |
| GANAB_HUMAN | GANAB | Neutral alpha-glucosidase AB | 1288 | 107263 | -6.67 | 🡻 | 6 | Energy Metabolism | Hydrolase activity |
| IDHP_HUMAN | IDH2 | Isocitrate dehydrogenase [NADP], mitochondrial | 371 | 51333 | -4.55 | 🡻 | 3 | Energy Metabolism | Catalytic activity |
| ALDOC_HUMAN | ALDOC | Fructose-bisphosphate aldolase C | 650 | 39830 | -3.45 | 🡻 | 3 | Energy Metabolism | Lyase activity |
| GLU2B_HUMAN | PRKCSH | Glucosidase 2 subunit beta | 455 | 60357 | -1.79 | 🡻 | 6 | Energy Metabolism | Glucosidase activity |
| PRDX1_HUMAN | PRDX1 | Peroxiredoxin-1 | 1256 | 22324 | 3.24 | 🡹 | 11 | Energy Metabolism | Peroxidase activity |
| AN32A_HUMAN | ANP32A | Acidic leucine-rich nuclear phosphoprotein 32 family member A | 812 | 28682 | -2.63 | 🡻 | 8 | Immune response | MHC class I receptor activity |
| PDIA1_HUMAN | P4HB | Protein disulfide-isomerase | 1034 | 57480 | -1.67 | 🡻 | 14 | Protein metabolism | Isomerase activity |
| IF4A2_HUMAN | EIF4A2 | Eukaryotic initiation factor 4A-II | 1548 | 46601 | -1.52 | 🡻 | 6 | Protein metabolism | Translation regulator activity |
| RL11_HUMAN | RPL11 | 60S ribosomal protein L11 | 578 | 20468 | -1.52 | 🡻 | 15 | Protein metabolism | Structural constituent of ribosome |
| RL32_HUMAN | RPL32 | 60S ribosomal protein L32 | 52 | 15964 | 10 | 🡹 | 3 | Protein metabolism | Structural constituent of ribosome |
| RL8_HUMAN | RPL8 | 60S ribosomal protein L8 | 509 | 28235 | 10 | 🡹 | 8 | Protein metabolism | Structural constituent of ribosome |
| RL19_HUMAN | RPL19 | 60S ribosomal protein L19 | 688 | 23565 | 2.68 | 🡹 | 7 | Protein metabolism | Structural constituent of ribosome |
| EF1G_HUMAN | EEF1G | Elongation factor 1-gamma | 1290 | 50429 | 1.72 | 🡹 | 12 | Protein metabolism | Translation regulator activity |
| PSB4_HUMAN | PSMB4 | Proteasome subunit beta type-4 | 284 | 29243 | 2.77 | 🡹 | 4 | Protein metabolism | Ubiquitin-specific protease activity |
| RL37_HUMAN | RPL37 | 60S ribosomal protein L37 | 66 | 11299 | 1.78 | 🡹 | 3 | Protein metabolism | Structural constituent of ribosome |
| TCPH_HUMAN | CCT7 | T-complex protein 1 subunit eta | 745 | 59842 | 3.96 | 🡹 | 5 | Protein metabolism | Chaperone activity |
| UBB_HUMAN | UBB | Polyubiquitin-B | 1068 | 25762 | 1.98 | 🡹 | 21 | Protein metabolism | Protein binding |
| PRS6B_HUMAN | PSMC4 | 26S protease regulatory subunit 6B | 596 | 47451 | -10.00 | 🡻 | 3 | Protein metabolism | Ubiquitin-specific protease activity |
| MCA2_HUMAN | AIMP2 | Aminoacyl tRNA synthase complex-interacting multifunctional protein 2 | 333 | 35668 | -9.09 | 🡻 | 4 | Protein metabolism | Translation regulator activity |
| SERPH_HUMAN | SERPINH1 | Serpin H1 | 1018 | 46525 | 2.01 | 🡹 | 4 | Protein metabolism | Heat shock protein activity |
| TCPB_HUMAN | CCT2 | T-complex protein 1 subunit beta | 2682 | 57794 | -6.25 | 🡻 | 10 | Protein metabolism | Chaperone activity |
| EF2_HUMAN | EEF2 | Elongation factor 2 | 3461 | 96246 | -5.88 | 🡻 | 16 | Protein metabolism | Translation regulator activity |
| PDIA3_HUMAN | PDIA3 | Protein disulfide-isomerase A3 | 1696 | 57146 | -4.55 | 🡻 | 7 | Protein metabolism | Isomerase activity |
| RS15A_HUMAN | RPS15A | 40S ribosomal protein S15a | 431 | 14944 | -4.35 | 🡻 | 4 | Protein metabolism | Structural constituent of ribosome |
| GRP78_HUMAN | HSPA5 | 78 kDa glucose-regulated protein | 3761 | 72402 | -3.85 | 🡻 | 11 | Protein metabolism | Chaperone activity |
| RL17_HUMAN | RPL17 | 60S ribosomal protein L17 | 149 | 21611 | -3.85 | 🡻 | 4 | Protein metabolism | Structural constituent of ribosome |
| PPIA_HUMAN | PPIA | Peptidyl-prolyl cis-trans isomerase A | 2202 | 18229 | -3.70 | 🡻 | 8 | Protein metabolism | Isomerase activity |
| PDIA6_HUMAN | PDIA6 | Protein disulfide-isomerase A6 | 1412 | 48490 | -3.70 | 🡻 | 3 | Protein metabolism | Isomerase activity |
| NPM_HUMAN | NPM1 | Nucleophosmin | 837 | 32726 | -3.03 | 🡻 | 5 | Protein metabolism | Chaperone activity |
| DJB11_HUMAN | DNAJB11 | DnaJ homolog subfamily B member 11 | 60 | 40774 | -2.50 | 🡻 | 3 | Protein metabolism | Chaperone activity |
| HSP7C_HUMAN | HSPA8 | Heat shock cognate 71 kDa protein | 4803 | 71082 | -2.38 | 🡻 | 24 | Protein metabolism | Heat shock protein activity |
| HSP76_HUMAN | HSPA6 | Heat shock 70 kDa protein 6 | 2045 | 71440 | -2.33 | 🡻 | 5 | Protein metabolism | Heat shock protein activity |
| UCHL1_HUMAN | UCHL1 | Wee1-like protein kinase | 696 | 25151 | -2,27 | 🡻 | 3 | Protein metabolism | Ubiquitin-specific protease activity |
| RS8_HUMAN | RPS8 | 40S ribosomal protein S8 | 1225 | 24475 | -2.00 | 🡻 | 3 | Protein metabolism | Structural constituent of ribosome |
| GRP75_HUMAN | HSPA9 | Stress-70 protein, mitochondrial | 1310 | 73920 | -1.75 | 🡻 | 4 | Protein metabolism | Chaperone activity |
| PDIA4_HUMAN | PDIA4 | Protein disulfide-isomerase A4 | 597 | 73229 | -1.72 | 🡻 | 4 | Protein metabolism | Chaperone activity |
| EF1A2_HUMAN | EEF1A2 | Elongation factor 1-alpha 2 | 1146 | 50780 | 2.27 | 🡹 | 15 | Protein metabolism | Translation regulator activity |
| CH60_HUMAN | HSPD1 | 60 kDa heat shock protein, mitochondrial | 2786 | 61187 | 2.32 | 🡹 | 19 | Protein metabolism | Heat shock protein activity |
| PSA1_HUMAN | PSMA1 | Proteasome subunit alpha type-1 | 421 | 29822 | 2.49 | 🡹 | 5 | Protein metabolism | Peptidase activity |
| RS3A_HUMAN | RPS3A | 40S ribosomal protein S3a | 1717 | 30154 | 2.49 | 🡹 | 8 | Protein metabolism | Structural constituent of ribosome |
| RS18_HUMAN | RPS18 | 40S ribosomal protein S18 | 450 | 17708 | 2.55 | 🡹 | 6 | Protein metabolism | Structural constituent of ribosome |
| RL10_HUMAN | RPL10 | 60S ribosomal protein L10 | 255 | 25044 | 3.22 | 🡹 | 9 | Protein metabolism | Structural constituent of ribosome |
| RL13_HUMAN | RPL13 | 60S ribosomal protein L13 | 603 | 24304 | 3.24 | 🡹 | 7 | Protein metabolism | RNA binding |
| RS13_HUMAN | RPS13 | 40S ribosomal protein S13 | 240 | 17212 | 3.24 | 🡹 | 8 | Protein metabolism | Structural constituent of ribosome |
| RS16_HUMAN | RPS16 | 40S ribosomal protein S16 | 139 | 16549 | 3.59 | 🡹 | 6 | Protein metabolism | Structural constituent of ribosome |
| PSB1_HUMAN | PSMB1 | Proteasome subunit beta type-1 | 457 | 26700 | 4.20 | 🡹 | 3 | Protein metabolism | Ubiquitin-specific protease activity |
| TBCB_HUMAN | TBCB | Tubulin-folding cofactor B | 745 | 27594 | 4.26 | 🡹 | 4 | Protein metabolism | Chaperone activity |
| RS2_HUMAN | RPS2 | 40S ribosomal protein S2 | 159 | 31590 | 4.43 | 🡹 | 4 | Protein metabolism | Structural constituent of ribosome |
| TCP4_HUMAN | SUB1 | Activated RNA polymerase II transcriptional coactivator p15 | 225 | 14386 | -1.52 | 🡻 | 4 | Reg. of nucleic acid metab | Transcription factor activity |
| PABP1_HUMAN | PABPC1 | Polyadenylate-binding protein 1 | 936 | 70854 | 10 | 🡹 | 5 | Reg. of nucleic acid metab | RNA binding |
| RU2A_HUMAN | SNRPA1 | U2 small nuclear ribonucleoprotein A' | 708 | 28512 | 10 | 🡹 | 5 | Reg. of nucleic acid metab | RNA binding |
| IF4A3_HUMAN | EIF4A3 | Eukaryotic initiation factor 4A-III | 409 | 47126 | -1.52 | 🡻 | 6 | Reg. of nucleic acid metab | Molecular function unknown |
| PHB2_HUMAN | PHB2 | Prohibitin-2 | 2202 | 33276 | 3.60 | 🡹 | 4 | Reg. of nucleic acid metab | Transcription regulator activity |
| H4_HUMAN | HIST1H4A | Histone H4 | 898 | 11360 | 2.87 | 🡹 | 6 | Reg. of nucleic acid metab | DNA binding |
| NP1L1_HUMAN | NAP1L1 | Nucleosome assembly protein 1-like 1 | 517 | 45631 | -4.35 | 🡻 | 4 | Reg. of nucleic acid metab | DNA binding |
| HNRPF_HUMAN | HNRNPF | Heterogeneous nuclear ribonucleoprotein F | 1418 | 45985 | -3.85 | 🡻 | 5 | Reg. of nucleic acid metab | Ribonucleoprotein |
| PSPC1_HUMAN | PSPC1 | Paraspeckle component 1 | 315 | 58820 | -3.70 | 🡻 | 3 | Reg. of nucleic acid metab | RNA binding |
| NUCL_HUMAN | NCL | Nucleolin | 578 | 76625 | -3.57 | 🡻 | 4 | Reg. of nucleic acid metab | RNA binding |
| SFRS7_HUMAN | SRSF7 | Serine/arginine-rich splicing factor 7 | 650 | 27578 | -3.33 | 🡻 | 3 | Reg. of nucleic acid metab | RNA binding |
| HNRPM_HUMAN | HNRNPM | Heterogeneous nuclear ribonucleoprotein M | 1387 | 77749 | -3.23 | 🡻 | 10 | Reg. of nucleic acid metab | Ribonucleoprotein |
| DDX5_HUMAN | DDX5 | Probable ATP-dependent RNA helicase DDX5 | 2085 | 69618 | -3.13 | 🡻 | 11 | Reg. of nucleic acid metab | RNA binding |
| SFPQ_HUMAN | SFPQ | Splicing factor, proline- and glutamine-rich | 793 | 76216 | -2.56 | 🡻 | 6 | Reg. of nucleic acid metab | RNA binding |
| RALY_HUMAN | RALY | RNA-binding protein Raly | 254 | 32501 | -2.44 | 🡻 | 3 | Reg. of nucleic acid metab | RNA binding |
| U2AF1_HUMAN | U2AF1 | Splicing factor U2AF 35 kDa subunit | 305 | 28368 | -1.92 | 🡻 | 4 | Reg. of nucleic acid metab | RNA binding |
| FUBP1_HUMAN | FUBP1 | Far upstream element-binding protein 1 | 507 | 67690 | -1.72 | 🡻 | 3 | Reg. of nucleic acid metab | Transcription regulator activity |
| ROA2_HUMAN | HNRNPA2B1 | Heterogeneous nuclear ribonucleoproteins A2/B1 | 3369 | 37464 | 1.52 | 🡹 | 11 | Reg. of nucleic acid metab | Transcription factor binding |
| U2AF2_HUMAN | U2AF2 | Splicing factor U2AF 65 kDa subunit | 176 | 53809 | 1.59 | 🡹 | 3 | Reg. of nucleic acid metab | Spliceosomal catalysis |
| HNRPK_HUMAN | HNRNPK | Heterogeneous nuclear ribonucleoprotein K | 1880 | 51230 | 2.32 | 🡹 | 16 | Reg. of nucleic acid metab | Ribonucleoprotein |
| HNRPC_HUMAN | HNRNPC | Heterogeneous nuclear ribonucleoproteins C1/C2 | 626 | 33707 | 2.35 | 🡹 | 6 | Reg. of nucleic acid metab | RNA binding |
| HNRPG_HUMAN | RBMX | RNA-binding motif protein, X chromosome | 545 | 42306 | 2.41 | 🡹 | 6 | Reg. of nucleic acid metab | RNA binding |
| IF2B1_HUMAN | IGF2BP1 | Insulin-like growth factor 2 mRNA-binding protein 1 | 326 | 63759 | 2.50 | 🡹 | 3 | Reg. of nucleic acid metab | RNA binding |
| ROA3_HUMAN | HNRNPA3 | Heterogeneous nuclear ribonucleoprotein A3 | 1297 | 39799 | 2.55 | 🡹 | 7 | Reg. of nucleic acid metab | RNA binding |
| PTBP1_HUMAN | PTBP1 | Polypyrimidine tract-binding protein 1 | 2182 | 57357 | 3.24 | 🡹 | 4 | Reg. of nucleic acid metab | Ribonucleoprotein |
| PARK7_HUMAN | PARK7 | Protein DJ-1 | 477 | 20050 | 3.33 | 🡹 | 5 | Reg. of nucleic acid metab | RNA binding |
| H2B1A_HUMAN | HIST1H2BA | Histone H2B type 1-A | 333 | 14159 | 4.16 | 🡹 | 4 | Reg. of nucleic acid metab | DNA binding |
| H2B3B_HUMAN | HIST3H2BB | Histone H2B type 3-B | 531 | 13900 | 4.32 | 🡹 | 5 | Reg. of nucleic acid metab | DNA binding |
| RL18A_HUMAN | RPL18A | 60S ribosomal protein L18a | 465 | 21034 | 4.57 | 🡹 | 4 | Reg. of nucleic acid metab | RNA binding |
| H2B1J_HUMAN | HIST1H2BJ | Histone H2B type 1-J | 468 | 13896 | 5.30 | 🡹 | 4 | Reg. of nucleic acid metab | DNA binding |
| NUCKS_HUMAN | NUCKS1 | Nuclear ubiquitous casein and cyclin-dependent kinase substrate 1 | 75 | 27280 | 6.49 | 🡹 | 4 | Reg. of nucleic acid metab | DNA binding |
| SFXN1_HUMAN | SFXN1 | Sideroflexin-1 | 813 | 35881 | -5.00 | 🡻 | 3 | Transport | Transporter activity |
| GDIB_HUMAN | GDI2 | Rab GDP dissociation inhibitor beta | 935 | 51087 | -4.76 | 🡻 | 4 | Transport | Auxiliary transport protein activity |
